# The effect of colostrum intake on blood plasma proteome profile in newborn lambs: low abundance proteins

**DOI:** 10.1186/1746-6148-10-85

**Published:** 2014-04-05

**Authors:** Lorenzo Enrique Hernández-Castellano, André Martinho Almeida, Miguel Ventosa, Ana Varela Coelho, Noemí Castro, Anastasio Argüello

**Affiliations:** 1 Department of Animal Science, Universidad de Las Palmas de Gran Canaria, Arucas, Gran Canaria, Spain; 2 Instituto de Tecnología Química e Biologica, Universidade Nova de Lisboa, Oeiras, Portugal; 3 Instituto de Investigação Científica Tropical (IICT) & Centro Interdisciplinar de Investigação em Sanidade Animal (CIISA), Lisbon, Portugal; 4 Instituto de Biologia Experimental e Tecnológica, Oeiras, Portugal

## Abstract

**Background:**

Colostrum intake by newborn lambs plays a fundamental role in the perinatal period, ensuring lamb survival. In this study, blood plasma samples from two groups of newborn lambs (Colostrum group and Delayed Colostrum group) at 2 and 14 h after birth were treated to reduce the content of high abundance proteins and analyzed using Two-Dimensional Differential in Gel Electrophoresis and MALDI MS/MS for protein identification in order to investigate low abundance proteins with immune function in newborn lambs.

**Results:**

The results showed that four proteins were increased in the blood plasma of lambs due to colostrum intake. These proteins have not been previously described as increased in blood plasma of newborn ruminants by colostrum intake. Moreover, these proteins have been described as having an immune function in other species, some of which were previously identified in colostrum and milk.

**Conclusions:**

In conclusion, colostrum intake modified the low abundance proteome profile of blood plasma from newborn lambs, increasing the concentration of apolipoprotein A-IV, plasminogen, serum amyloid A and fibrinogen, demonstrating that colostrum is essential, not only for the provision of immunoglobulins, but also because of increases in several low abundance proteins with immune function.

## Background

The relationship between colostrum intake and newborn ruminant survival has been extensively characterized [1-4]. Colostrum is the first source of nutrition in neonatal ruminants, supplying not only nutrients, but having also a fundamental biological function, promoting immunoglobulin (Ig) transfer from the dam to the newborn. Moreover, colostrum has been described as having a mixture of diverse components, such as fat, lactose, vitamins and minerals that have a high nutritional importance [5]. However, beyond the nutritional function, colostrum contains a complex mixture of proteins that actively participate in the protection of the neonate against pathogens and other post-partum environmental challenges [6].

To date, a wide variety of colostrum and milk bioactive peptides and proteins have not only been linked to the passive immune transfer, such as lactoferrin [7], lactoperoxidase [8] or lysozymes [9], but also promoting gastrointestinal growth and development of the newborn, such as insulin-like growth factors (IGF-1 and IGF-2) or the transforming growth factor beta (TGF-BI and TGF-B2) [6,10].

As a consequence, colostrum intake and colostrum protein absorption play an essential role in passive immune transfer and ultimately in newborn survival [11,12]. However, the absorbance conditions for intact proteins decrease during the first 48 h after birth, therefore colostrum feeding must take place during this period [13]. It has been described how feeding newborn ruminants with colostrum, with a concentration of Igs that is insufficient results in high mortality rates and low productive performances, with negative consequences for the economic benefits of farmers and breeders and severe consequences to animal welfare [14,15].

Proteomics has been used to characterize protein changes in the transition from colostrum to milk in cattle [16,17]. Additionally, the study of low abundance proteins from different body fluids such as blood plasma, colostrum or milk is becoming increasingly relevant [16,18-21].

Despite the previous proteomic studies in colostrum and milk, it is still not fully known which proteins are absorbed or increased in lamb blood plasma as a result of colostrum intake. It is hypothesized that early colostrum intake modifies the proteome of newborn lamb blood plasma. For this reason, the aim of this study was to analyze blood plasma low abundance proteins from colostrum-fed lambs compared to colostrum-deprived lambs, during the first 14 h after birth, in order to identify these plasma protein changes. Results from this study will contribute to understand the importance of colostrum on passive immune transfer and the lamb immune system development.

## Results and discussion

In this experiment we have used an approach based on the analysis of the proteome of low abundant proteins in plasma from newborn lambs using the ProteoMiner® (Bio-Rad, Hercules, CA, USA) technology that allows the removal of the higher-abundance proteins in the plasma, particularly albumin, IgG and IgM, followed by a 2-DE DIGE analysis and protein identification using mass spectrometry. With reference to this topic, many methodologies can be found in the market, however several authors have observed excellent results using ProteoMiner® not only in the removal of high abundance proteins, but also in the high concentration and intensity of low abundance proteins [21,22].

During this study, no body weight differences were observed between groups (see Table 1). Moreover, no evidences of illness were detected during health status monitoring.

**Table 1:** BW and blood plasma IgG and IgM evolution in Colostrum (C) and Delayed Colostrum (DC) groups at 2 and 14 h after birth

	**Group**	**Time after birth (h)**	
		**2**	**14**
BW (kg)	C	4.17 ± 0.32	4.12 ± 0.17
	DC	4.26 ± 0.29	4.18 ± 0.34
IgG (mg/mL)	C	ND	7.406 ± 0.76
	DC	ND	ND
IgM (mg/mL)	C	ND	0.443 ± 0.08
	DC	ND	ND

ND means No detectable; BW means Body weight; IgG means Immunoglobulin G; IgM means Immunoglobulin.

We evaluated the levels of IgG and IgM in non-ProteoMiner® treated plasma samples in order to determine the presence or absence of colostrum proteins in both groups (C group and DC group) at the two studied times (2 and 14 h after birth). Results are shown also in Table 1 where the concentration of the two Igs in the two studied groups (C and DC groups) and at 2 and 14 h after birth is presented. At birth (2 h) animals from both groups had no detectable (ND) IgG concentration in blood. However, when both groups were compared at 14 h after birth, IgG concentration could be detected only in C group (7.406 mg/mL *vs.* ND in C and DC group, respectively). A similar pattern was observed when IgM concentrations were analyzed, with no detection at 2 h after birth in any of the experimental groups and being detected in C group, but not in the DC group, at 14 h after birth. Several authors have observed a similar evolution in Igs level, depending on the total amount of Igs present in colostrum, in newborn blood from lambs [23], calves [24,25] and goat kids [2,26]. As expected, these results confirm that the presence of colostrum IgG and IgM in the C group in blood at 14 h after birth is due to colostrum intake.

**Figure 1:**
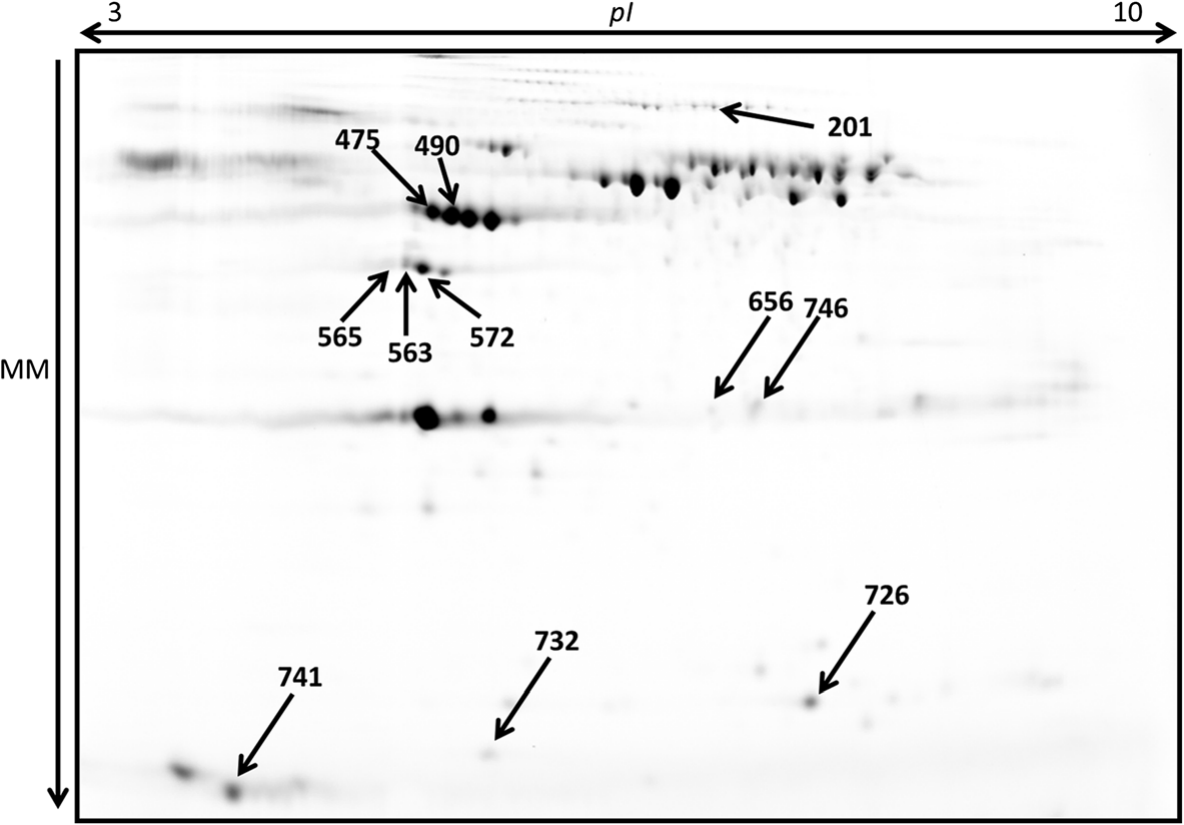
**Lamb plasma pool labelled with Cy2 used as a reference gel in gel analysis.** Protein extracts were processed using ProteoMiner® commercial kit and used in DIGE, where a total of 50 μg were loaded per CyDye. Spots showing differential expression are highlighted with arrows*. pI – Isoelectric point and M – Molecular Mass. *These spots were similar between groups (C and DC group) at 2 h after birth and did not increase in the DC group at 14 h. Moreover, these spots were found to increase when C and DC group were compared at 14 h.

As shown in Figure 1, a total of 11 spots showing over-expression in lambs at 14 h after birth were detected in the C group. These spot relative intensities were similar between groups (C and DC group) at 2 h after birth and did not increase in the DC group at 14 h (Table 2). Of these 11 spots, we were able to identify a total of 7 spots, as presented in Table 3. The spots were identified as apolipoprotein A-IV (spots 563,565 and 572), plasminogen (spot 201), serum amyloid A (spot 726) and fibrinogen gamma chain (spots 475 and 490). These proteins may play an important role either in the immune-system development or in the immune protection or even both at the early stages of life and will subsequently be described separately.

**Table 2:** Spots showing differential expression between at least two experimental groups (p < 0.05 and fold >1.3)

**Spot reference**	**C 2 h vs. DC 2 h**		**C 2 h vs. C 14 h**		**DC 2 h vs. DC 14 h**		**Average normalized volumes**				**Spot image**			
							**C group**		**DC group**		**C group**		**DC group**	
	**P-value**	**Fold**	**P-value**	**Fold**	**P-value**	**Fold**	**2 h**	**14 h**	**2 h**	**14 h**	**2 h**	**14 h**	**2 h**	**14 h**
490	0.690	1.0	<0.001	2.1	0.392	1.1	0.835	1.770	0.926	0.988				
201	0.214	1.2	<0.001	2.1	0.752	1.0	0.843	1.810	0.991	0.944				
565	0.356	1.5	0.002	1.3	0.356	1.7	0.835	1.122	0.853	0.927				
475	0.052	1.1	0.005	1.3	0.360	1.1	1.187	1.930	0.953	1.056				
572	0.105	1.3	0.007	3.0	0.056	1.5	0.832	2.531	0.858	1.019				
726	0.134	2.4	0.007	4.0	0.133	1.1	0.442	1.779	0.586	1.137				
563	0.255	1.5	0.008	1.3	0.217	1.8	0.891	1.116	0.845	0.980				

C 2 h and DC 2 h means Colostrum group and Delayed Colostrum group at 2 h after birth, respectively; C 14 h and DC 14 h means Colostrum group and Delayed Colostrum group at 14 h after birth.

**Table 3:** Mass spectrometry identification of differentially expressed proteins from lamb plasma

**Spot reference**	**Protein name**	**Accession number**	**Theoretical molecular mass (kDa)**	**Theoretical PI**	**Matched peptides**^**a**^		**Sequence coverage (%)**^**b**^	**Protein score**^**c**^
					**MS**	**MS/MS**		
490	Fibrinogen gamma-B chain	FIBG_BOVIN	50.8	5.5	7	5	16	361
201	Plasminogen (Fragment)	PLMN_SHEEP	38.6	7.5	7	-	23	86
565	Apolipoprotein A-IV	APOA4_BOVIN	42.9	5.3	12	1	26	183
475	Fibrinogen gamma-B chain	FIBG_BOVIN	50.8	5.5	8	4	15	332
572	Apolipoprotein A-IV	APOA4_BOVIN	42.9	5.3	17	8	35	738
726	Serum amyloid A	SAA_BOVIN	14.5	7.8	4	1	30	62
563	Apolipoprotein A-IV	APOA4_BOVIN	42.9	5.3	15	1	35	432

^a^Number of peptides, matching the identified protein, whose sequence differs in at least one amino acid residue; ^b^Percentage of the identified protein sequence covered by the matched peptides; ^c^Identification Score obtained with the Mowse algorithm. A result is considered to be significant when a score above 61 is attained.

### Apolipoprotein A-IV (Apo A-IV)

The metabolic function of apolipoprotein A-IV (Apo A-IV) has not been fully established, however it has been suggested that Apo A-IV plays an important role at early life, modulating the enterocyte lipid transport efficiency in fatty foods, namely colostrum [27]. For this reason, the intestinal synthesis and secretion of Apo A-IV increases during fat absorption [28]. Additionally, Apo A-IV has antioxidant properties, acts as a postprandial satiety signal, and reduces gastric acid secretion [27]. An increase in the expression of this protein in colostrum may play a role in the protection of the immunoglobulin molecule structure, reducing the gastric acid secretion in the stomach of the newborn lamb and increasing the total amount of intact Igs absorbed in the intestine.

Finally, Apo A-IV has been described as having an immunomodulatory effect against external agents (e.g. experimental induced colitis using dextran sulfate sodium) in mice [29]. Consequently, an increase in the blood plasma levels of Apo A-IV could also contribute to protect the newborn from infections at this early stage of life.

### Plasminogen (PLG)

This glycoprotein is the precursor of plasmin, a fibrinolytic enzyme that plays an important role in the dissolution of fibrin blood clots in order to prevent thrombosis [30,31]. Nevertheless, this protein has been identified not only in blood, but also in colostrum and milk [32]. In bovine, sheep and goat milk, plasmin and plasminogen forms are identical to those found in blood [33].

In addition to its main role in the dissolution of fibrin blood clots, plasminogen is structurally similar to Apo A-IV [34]. Apo A-IV, as with other Apo A proteins, has the capacity to bind fibrin and proteins of endothelial cells and monocytes, and therefore may inhibit plasminogen binding and plasmin generation [35]. The presence of this protein in colostrum and the increase of this protein in blood plasma could thwart the Apo A-IV to bind fibrin in newborn lambs.

Additionally, plasminogen has immune activity, as it contributes to neutrophil migration to an infection site [36]. An increase of this protein expression in plasma promotes the neutrophil migration in blood and therefore may contribute to the immune response against potential infections in the newborn lamb. In agreement with these findings, Theodorou et al. [37] found an increase of plasminogen concentration in blood and milk during acute mastitis in lactating dairy ewes. Therefore the colostrum intake seems to be an important factor that increases plasminogen concentration in blood at this stage of life.

Plasminogen participates also in the regulation of cellular apoptosis [38], specifically the apoptosis of adherent cells induced by disruption of integrin-mediated cell-matrix interactions. This has been described under specific physiological conditions, such as the involution of the mammary gland after lactation and the renewal of intestinal epithelial cells. The apoptotic processes in the latter tissue is of important relevance in the absorption of Igs by newborn ruminants during the first hours after birth, as described by Castro-Alonso et al. [39]. Therefore, the increase of this protein in blood may delay the decrease of the apoptosis rate of intestinal epithelial cells during the early stage of life, increasing the available time for colostrum components absorption, including both Igs and other proteins.

### Serum amyloid a (SAA)

Serum Amyloid A (SAA) is normally found in different isoforms and complexes with lipoproteins, while its plasma concentration vary depending on the species [40]. It is an apolipoprotein that takes part in the acute phase of inflammation [41-43] and represents one of the most conserved proteins among mammals supporting the premise that it has a basic and essential role in the innate immune system. However, this protein has also been identified in colostrum of several species, such as human [44], horse, cattle and sheep [45,46]. The importance of it in inflammatory processes has been fully monitored, showing that the circulating concentration of SAA protein is increased by 1000-fold within 24 to 48 h after infection/inflammation from a basal level of 0.82 ± 0.53 μg/mL [47].

The SAA protein has numerous pro-inflammatory actions: it works as a chemoattractant to neutrophils, monocytes, and T lymphocytes, causing leukocyte infiltration and promoting neutrophil adhesion to endothelial cells [48-50], stimulating neutrophils and monocytes to release not only cytokines [51,52], but also matrix metalloproteinases [53]. According to He et al. [54], these findings suggest a key function for SAA not only in the establishment, but also in the maintenance of inflammation, meaning that newborn lambs fed with colostrum at this early stage of life reportedly have a clear advantage, increasing this protein level in blood, and consequently producing a more efficient immune status.

### Fibrinogen gamma chain (FGG)

The fibrinogen gamma chain (FGG) is one of the three components of fibrinogen which is the precursor of fibrin, the most abundant component in blood clots. However, this protein also has a defensive function, having been demonstrated that fibrinogen concentration increases during acute-phase reactions [55,56]. Moreover, Yamada et al. [57] studied differences in low abundance proteins between bovine colostrum and milk, showing that some of them were only present in colostrum, such as fibrinogen, which could explain the plasma increase of this protein in animals that were fed with it. Additionally, several authors have described that fibrinogen can bind to integrins [58,59], that are normally expressed on cells of the immune system, such as CD11b^+^/CD18^+^ monocytes. The CD11b^+^/CD18^+^ integrin receptor (αMβ2, Mac-1, complement receptor 3) is a member of the β2 integrin family, which is in turn expressed on monocytes and macrophages. When fibrinogen binds to CD11b^+^/CD18^+^, integrin causes an extensive array of cell signaling responses, namely the activation of the nuclear factor kappa-light-chain-enhancer of activated B cells (NF-κB) and mitogen-activated protein kinase (MAPK)/phosphatidylinositol 3-kinase (PI3K). This data indicates that fibrinogen can function not only as a substrate in the clotting cascade, but also as an important effector during the evolution of the innate immune response [60]. Therefore, intake of colostrum and the subsequent increase of fibrinogen in lamb blood plasma may benefit the newborn immune system efficiency.

## Conclusions

In conclusion, early colostrum intake produced an increase of non-immunoglobulin proteins in lamb blood plasma, such as apoliprotein A-IV, plasminogen, serum amyloid A and fibrinogen. These proteins have reported immune functions in other species, suggesting that colostrum provides not only Igs, but also non-immunoglobulin proteins. These proteins play a fundamental role in the activation and attraction of immune cells, the apoptosis rate of the enterocytes and the low gastric secretion, among other roles. The results of this work contribute information about proteins with immune function that are increased after colostrum intake. High plasma concentrations of these proteins may decrease lamb mortality and increase the economic benefit for farmers. In the future, further proteomic studies will be necessary in order to increase the general knowledge about the role of colostrum in the passive immune transfer. Such studies could consist in the fully qualitative characterization of proteins present in colostrum, as well as samples from C and DC group at 2, 14 and 26 h. Moreover, a quantitative study could be performed on samples from both groups (C and DC group) at 2, 14 and 26 h in order to detect if lambs, receiving a delayed colostrum meal (14 h) are able to reach a similar protein concentration than lambs fed with colostrum after birth (2 h) at 26 h after birth.

## Methods

The experiment was approved by the ethics committee of the Faculty of Veterinary of the *Universidad de Las Palmas de Gran Canaria.*

### Samples collection

The Canarian dairy sheep breed was used for this experiment. This breed is a high yield dairy breed (1.8 L/d) with a lactation period of 180-200 days [61]. Ewes were fed with corn, soy 44 (crude protein 44 per cent), dehydrated lucerne, dehydrated beetroot, lucerne hay and a vitamin-mineral supplement in accordance with the guidelines issued by the *Institut Nationale de la Recherche Agronomique* (Paris, France) [62]. Two groups of 6 newborn male lambs each from single births were used in this experiment. The experiment took place at the experimental farm of the Veterinary Faculty of the Universidad de Las Palmas de Gran Canaria (Canary Islands, 28° 8' 20.66" N, 15° 30' 24.97" W, Spain) in spring. Animals were fed with sheep colostrum at different time points. One group (termed Colostrum group; C group) received two colostrum meals, at 2 and 14 h after birth. The other group (termed Delayed Colostrum group; DC group) was not fed with colostrum at 2 h after birth, but received one colostrum meal at 14 h after birth in order to ensure the survival of these animals.

Comparative analysis of different fluids (such as colostrum and plasma samples) using 2-D gels is not meaningful. Instead one group did not receive colostrum (DC group) during the experimental period (from birth to 14 hours after birth before feeding). Plasma from this group without colostrum proteins was compared with plasma from lambs that received a colostrum meal (C group) at 2 hours after birth. In order to ensure the survival of lambs, each animal (from both groups) was also fed at 24 h after birth. The total volume of colostrum was equivalent to 4 g of IgG/kg of BW as previously suggested in colostrum immune studies [26,63].

The colostrum source was from a frozen pool with containing Immunoglobulin G (IgG) at a concentration of 64.74 mg/mL. Blood samples from both groups were collected before feeding at 2 and 14 h after birth. The only difference between the groups was the colostrum administration at 2 h after birth. Tubes with K- ethylenediaminetetraacetic acid (EDTA) were used for blood sampling. After centrifugation, the plasma sample was frozen at -80°C until further analysis. During this experiment, lambs were accommodated in artificial rearing rooms, providing at least 0.3 m^2^ floor space per lamb. Each room had central heating conferring a room temperature of approximately 20°C and followed standard commercial procedures used in the Canary Islands.

Blood Plasma IgG and IgM was analysed to determine the presence of colostrum proteins in lamb plasma from both groups (C group and DC group) at the two studied times (2 and 14 h after birth). To determine plasma IgG and IgM concentration, a commercial ELISA kit (Bethyl Laboratories, Montgomery, TX, USA) was used, setting a purified sheep IgG and IgM as standard reference.

Statistical analyses of IgG and IgM were performed using SAS, Version 9.00 (SAS Institute Inc., Cary, NC). The SAS PROC MIXED procedure for repeated measurements was used to evaluate the effect of colostrum intake (C group *vs.* DC group) at 2 and 14 h after birth. A Bonferroni’s test was used to evaluate differences between groups.

### Animal weighting and health status

Lambs were weighed on a digital scale before each blood extraction (2 and 14 h after birth). Animal health status was monitored during the experimental period (for diarrhea, parasites or fever) and animals were found to be healthy throughout the experimental period.

### Sample treatment for analysis

The proteomic assays were carried out at the *Instituto de Tecnología Química e Biológica* (Oeiras, Portugal). In order to reduce the high-abundance proteins present in blood plasma (albumin and immunoblogulins), plasma samples (200 μL) were processed with a Protein Enrichment Kit (ProteoMiner® Bio-Rad, Hercules, CA, USA) following the manufacturer’s instructions. Samples were subsequently desalted with 2D-Clean-up® kit (GE Healthcare, Piscataway, NJ, USA) and quantified with 2D-Quant® kit (GE Healthcare, Piscataway, NJ, USA) following manufacturer’s instructions.

### Two-dimensional differential in Gel electrophoresis (DIGE)

For each DIGE gel, 50 μg of each treated sample was labelled with Cy3 or Cy5 cyanine dyes (GE Healthcare, Piscataway, NJ, USA), whilst the internal standard pool, created from an equal amount of protein from each studied sample, was labelled with Cy2 dye (see Additional file 1). After labelling, samples were mixed with 7 M urea, 2 M thiourea, 4% (w/v) CHAPS, 2% (w/v) DTT , 2% (v/v) ampholytes and 0.04% bromophenol blue solution (1%) up to a final volume of 150 μL. Immobiline DryStrips pH 3*-*10 and 24 cm length (GE Healthcare, Piscataway, NJ, USA) were passively rehydrated with 450 μL of rehydration buffer (7 M urea, 2 M thiourea, 4% (w/v) CHAPS and 0.04% bromophenol blue solution (1%)) for 6 h at room temperature. Isoelectric focusing was performed with an Ettan IPGphor 3 Isoelectric Focusing System coupled to a Manifold strip holding system (GE Healthcare, Piscataway, NJ, USA) following the program: 150 V for 3 h, 300 V for 3 h, a gradient of 1000 V for 6 h, a gradient of 10.000 V for 1 h and 10.000 V for 3 h. Subsequently, strips were equilibrated with 50 mM Tris*-*HCl pH 8.8, 6 M urea, 30% (v/v) glycerol, 2% (w/v) SDS and 0.02% bromophenol blue solution (1%), in two steps of 15 min with 1% (w/v) DTT and 2.5% (w/v) iodoacetamide, respectively.

After equilibration, proteins were separated in the second dimension using 12.5% polyacrylamide gels on an Ettan Dalt Six electrophoresis system (GE Healthcare, Piscataway, NJ, USA) using the running conditions recommended by the manufacturer (1 W/gel for 1 h and 2 W/gel for 14-16 h at 12°C) and using low-florescence glass plates.

Each DIGE gel was scanned with a Fluorescent Image Analyzer (Fujifilm FLA-5100, Fujifim, Tokyo, Japan), using preferred excitation/emission wavelengths for Cy2, Cy3, and Cy5 of 488/520, 532/580, and 633/670 nm, respectively, generating images that were used in gel analysis.

### Image analysis

In order to detect differentially expressed proteins, gels were analysed using Progenesis SameSpots software (Nonlinear Dynamics, Newcastle upon Tyne, UK) following the manufacturer’s instructions for DIGE gels. Spots with p < 0.05 and an intensity of at least 1.3 fold higher were considered to have significantly different expression levels.

### Visible gel staining, spot excision and digestion

In order to excise the selected spots, DIGE gels were stained with Coomassie Brilliant Blue G-250 as previously described [64]. Spots were then manually excised for individual in-gel digestion using trypsin [64]. Briefly, spots were washed with 30 μL of water for 30 minutes, washed in acetonitrile (50%), reduced with 10 mM DTT at 56°C for 45 minutes, alkylated with 55 mM iodoacetamide for 30 minutes, washed in acetonitrile (100%) and vacuum dried (SpeedVac®, Thermo Fisher Scientific, Waltham, MA, USA). Gel pieces were rehydrated with a digestion buffer (50 mM NH_4_HCO_3_ buffer) containing trypsin (Promega, Madison, WI, USA) and incubated overnight at 37°C. The digestion buffer containing peptides was acidified with formic acid, desalted and concentrated using C8 microcolumns (POROS R2®, Applied Biosystems, Foster City, CA, USA), as described [64].

### Protein identification

Protein identification was conducted as described [65]. Briefly, protein identification was conducted using MALDI-TOF–TOF data acquired with an Applied Biosystem 4800 Proteomics Analyzer (Applied Biosystems, Foster City, CA, USA) in both MS and MS/MS mode. Positively charged ions were analysed in the reflectron mode over the m/z range of 800–3500 Da. Each MS spectrum was obtained in a result independent acquisition mode with a total of 800 laser shots per spectra and a fixed laser intensity of 3500 V, being externally calibrated using des-Arg-Bradykinin (904.468 Da), angiotensin 1 (1296.685 Da), Glu-Fibrinopeptide B (1570.677 Da), ACTH (1–17) (2093.087 Da), and ACTH (18–39) (2465.199) (Calibration Mix from Applied Biosystems). Fifteen best precursors from each MS spectrum were selected for MS/MS analysis. MS/MS analyses were performed using CID (Collision Induced Dissociation) assisted with air, using a collision energy of 1 kV and a gas pressure of 1 × 106 Torr. Two thousand laser shots were collected for each MS/MS spectrum using a fixed laser intensity of 4500 V. The S/N ratio was set at 20 as recommended by manufacturer. Raw data was generated by the 4000 Series Explorer Software v3.0 RC1 (Applied Biosystems, Foster City, CA, USA) and all contaminant m/z peaks originating from human keratin, trypsin autodigestion, or matrix were included in the exclusion list used to generate the peptide mass list used in the database search.

The generated mass spectra were used to search the NCBI predicted protein database, setting a taxonomical restriction (mammal database). Searches were conducted using Mowse from MASCOT-demon 2.1.0 Software (Matrix-Science) algorithm. Protein identifications were accepted if protein score was above a threshold of 95% (p < 0.05). The interpretation of the combined MS + MS/MS data was carried out using the GPS Explorer Software (Version 3.5, Applied Biosystems, Foster City, CA, USA), using the following parameters: missed-cleavage, one; peptide tolerance, 50 ppm; fragment mass tolerance, 0.25 Da; fixed modification, carbamidomethylation of cysteine; and variable modification, methionine oxidation. From the predicted protein database, the theoretical molecular mass and pI of the identified proteins was obtained using the Expasy Mw/pI Tool (http://www.expasy.org/tools/pi_tool.html). The identified proteins were only considered if a MASCOT protein score above 61 (p < 0.05) was obtained.

## Competing interest

The authors declare that they have no competing interests.

## Authors’ contributions

LEHC carried out all sample collection and experimental work and wrote the manuscript. AMA and AVC coordinated and supervised all proteomic assays and manuscript preparation. MV carried protein identifications. NC supervised and advised on scientific content of the manuscript and critical revision of the text. AA designed, coordinated and supervised the study and manuscript preparation. All authors read and approved the final manuscript.

## Supplementary Material

Additional file 1 **DIGE experimental design.** A means Colostrum group at 2 hours after birth; B means Colostrum group at 14 hours after birth; X means Delayed Colostrum group at 2 hours after birth; Z means Delayed Colostrum group at 14 hours after birth. Animals from Colostrum group were numbered from 1 to 6. Animals from Delayed Colostrum group were numbered from 7 to 12. Click here for file
