# Supplementary material for: The effect of colostrum intake on blood plasma proteome profile in newborn lambs: low abundance proteins
Source: BMC Vet Res. 2014 Apr 5;10:85. doi: 10.1186/1746-6148-10-85 (PMC4108057; doi:10.1186/1746-6148-10-85)
Supplement: Additional file 1 — DIGE experimental design. A means Colostrum group at 2 hours after birth; B means Colostrum group at 14 hours after birth; X means Delayed Colostrum group at 2 hours after birth; Z means Delayed Colostrum group at 14 hours after birth. Animals from Colostrum group were numbered from 1 to 6. Animals from Delayed Colostrum group were numbered from 7 to 12. [file 1746-6148-10-85-S1.docx]

|  | Cy 5 | Cy 3 | Cy 2 |
| --- | --- | --- | --- |
| Gel 1 | A1 | Z7 | Pool of all samples involved in this experiment |
| Gel 2 | A2 | Z8 |  |
| Gel 3 | X9 | Z9 |  |
| Gel 4 | X10 | A3 |  |
| Gel 5 | B3 | A4 |  |
| Gel 6 | B4 | X7 |  |
| Gel 7 | B5 | X8 |  |
| Gel 8 | Z10 | B1 |  |
| Gel 9 | Z11 | B2 |  |
| Gel 10 | Z12 | B6 |  |
| Gel 11 | A5 | X12 |  |
| Gel 12 | X11 | A6 |  |

### Additional file 1 – DIGE experimental design

A means Colostrum group at 2 hours after birth; B means Colostrum group at 14 hours after birth; X means Delayed Colostrum group at 2 hours after birth; Z means Delayed Colostrum group at 14 hours after birth. Animals from Colostrum group were numbered from 1 to 6. Animals from Delayed Colostrum group were numbered from 7 to 12.
